# Adenomyosis: An Update Concerning Diagnosis, Treatment, and Fertility

**DOI:** 10.3390/jcm13175224

**Published:** 2024-09-03

**Authors:** Aikaterini Selntigia, Pietro Molinaro, Silvio Tartaglia, Antonio Pellicer, Daniela Galliano, Mauro Cozzolino

**Affiliations:** 1 IVIRMA Global Research Alliance, IVIRMA Roma, 00169 Rome, Italy; aikaterini.selntigia@ivirma.com (A.S.); a.pellicer@ivirma.com (A.P.);; 2 Department of Biomedicine and Prevention, University of Rome Tor Vergata, Viale Montpellier 1, 00133 Rome, Italy; silvio.tartaglia@hotmail.it; 3 Department of Women, Children, and Public Health Sciences, Fondazione Policlinico Universitario A Gemelli IRCCS, 00168 Rome, Italy

**Keywords:** adenomyosis, diagnosis, treatment, IVF, reproductive outcomes

## Abstract

This review article aims to summarize current tools used in the diagnosis of adenomyosis with relative pharmacological and surgical treatment and to clarify the relative association between adenomyosis and infertility, considering the importance of an accurate diagnosis of this heterogeneous disease. Among different reported concepts, direction invagination of gland cells from the basalis endometrium deep into the myometrium is the most widely accepted opinion on the development of adenomyosis. Adenomyosis has been increasingly identified in young women with pain, AUB, infertility, or no symptoms by using imaging techniques such as transvaginal ultrasound and magnetic resonance. Furthermore, adenomyosis often coexists with other gynecological conditions, such as endometriosis and uterine fibroids, increasing the heterogeneity of available data. However, there is no agreement on the definition and classification of adenomyotic lesions from both the histopathology and the imaging points of view, and diagnosis remains difficult and unclear. A standard, universally accepted classification system needs to be implemented to improve our understanding and inform precise diagnosis of the type of adenomyosis. This could be the key to designing RCT studies and evaluating the impact of adenomyosis on quality of life in terms of menstrual symptoms, fertility, and pregnancy outcome, given the high risk of miscarriage and obstetric complications.

## 1. Introduction

Adenomyosis is a common and benign gynecology condition that involves the uterus [1]. It is characterized by the infiltration of the myometrium by endometrial tissue (both glands and stroma) and is typically associated with hyperplasia of the smooth muscle tissue [1,2]. The prevalence of the disease ranges from 12 to 58% of women during their reproductive years, depending on the population heterogeneity examined in different studies and the absence of standard diagnostic criteria [3]. Among infertile women, the prevalence seems to be higher (24.4%) due to the older age or co-occurrence of endometriosis [4]. Although the pathogenesis of adenomyosis remains unclear, the most accepted theory attributes the origin of adenomyosis to the invagination of the endometrium basalis into the myometrium as a consequence of disrupted boundaries [5], which also relates to Sampson’s hypothesis on the origin of endometriosis. The trigger of the injury-repair process is still unclear; however, hyperestrogenism may cause uterine contraction and subsequent injury of the junctional zone [6]. Endometriosis and adenomyosis are associated with a wide spectrum of symptoms and may present various histopathological transformations, such as the presence of hyperplasia, atypia, and malignant transformation occurring under the influence of local inflammatory, vascular, and hormonal factors and by the alteration of tumor suppressor proteins and the inhibition of cell apoptosis, with an increased degree of lesion proliferation. Proinflammatory/vascular/hormonal changes trigger adenomyosis progression and the onset of cellular atypia and malignant transformation, exacerbating symptoms, especially local pain and vaginal bleeding. These triggers may represent future therapeutic targets [7].

A study on DNA from biopsy of uterine specimens with adenomyosis concluded that the epithelial cells of adenomyosis originate from the adjacent eutopic endometrium [8]. A follow-up single-cell RNA sequencing study confirmed that the stromal cell component of adenomyosis also originated from the eutopic endometrium [9]. These two studies support the long-held view that adenomyotic tissue originates from the adjacent basal endometrium via its entrapment in the myometrium [10]. The menstruation process, itself, may increase the risk of the entrapment of fragments of the basal layer within the myometrium. Repetitious menstrual episodes imply reiterative tissue hypoxia, necrosis, myometrial contractions, angiogenesis, and regenerative processes [9,11]. These events may disrupt the endo-myometrial junction, favoring the intra-myometrial invagination of basal endometrium fragments characterized by somatic mutations and conferring specific advantages for the development of adenomyosis [9].

Multiparity represents a risk factor for adenomyosis, probably caused by the displaced endometrium due to the hormonal environment of pregnancy or the mechanical action of the delivery [3]. As a consequence of similar mechanisms, women who report one or more miscarriages are more likely to present this condition, as uterine surgical procedures are also considered risk factors for adenomyosis [12]. Curiously, the incidence of adenomyosis is lower in daily smokers, which is related to lower levels of estrogens [12]. Exposure to estrogens can also explain the higher incidence in older women [4]. Symptomatology is often represented by hypermenorrhea, abnormal uterine bleeding, secondary dysmenorrhea, and chronic pelvic pain, although adenomyosis may often be asymptomatic and diagnosticated during a routine examination or diagnostic workup of couples with infertility [13,14]. Despite the high prevalence of adenomyosis during the reproductive life span, the role that it plays in fertility and reproduction remains rather unclear. Although it seems that it may not affect implantation, women with adenomyosis seem to have an increased risk of miscarriage [14,15]. In addition, adenomyosis significantly increases the risk of developing preeclampsia and small for gestational age (SGA) [16]. The impact of adenomyosis on reproductive outcomes highlights the importance of proper diagnosis and classification of the disease and establishing the most appropriate medical treatment to avoid or reduce the consequences during pregnancy. This narrative review, which includes several articles related to this topic, aims to systematize the diagnosis, treatments, and IVF outcomes of women with adenomyosis.

## 2. Diagnosis of Adenomyosis

For more than a century after adenomyosis was first described, the diagnostic gold standard was histological post-hysterectomy confirmation. Since then, advancements in transvaginal ultrasound (TVS) imaging and Magnetic Resonance Imaging (MRI) have led to early diagnosis. Studies have demonstrated the high sensitivity and specificity of two- and three-dimensional (2D-3D) TVS compared to MRI and/or histologic examinations, with findings ranging from 75 to 88% and 67 to 93%, respectively [17,18,19,20,21,22,23]. Routine pelvic evaluation with a conventional 2D transvaginal probe can identify signs of adenomyosis [24]. At the same time, these ultrasound criteria facilitate easily accessible diagnosis at an early stage with lower cost [25].

### 2.1. Ultrasound Diagnosis

Nowadays, 2D and 3D TVS offer the possibility of a precise description and diagnosis of adenomyosis. In 2022, the Morphological Uterus Sonographic Assessment (MUSA) group [26] revised the previous 2015 consensus [24] and indicated direct and indirect signs for the diagnosis of adenomyosis (Table 1). This new classification outlines the following direct diagnostic signs of adenomyosis:

1. Myometrial cysts and intramyometrial cystic formations of any size (presence of a hyperechogenic halo is not mandatory). The contents could be anechoic, low-level, ground glass or of mixed echogenicity and may be surrounded by a hyperechogenic rim. Doppler can be used for differential diagnosis between blood vessels and myometrial cysts;
2. Hyperechogenic islands with intramyometrial hyperechogenic areas within the myometrium that have no connection with the endometrium (regular, irregular, or ill-defined);
3. Echogenic subendometrial lines and buds with hyperechogenic subendometrial lines or buds perpendicular to the endometrial cavity in a continuum with the endometrium, disrupting the junctional zone (JZ). On the other hand, the diagnosis of adenomyosis remains uncertain in cases of the presence of only indirect signs, including uterine wall asymmetry, translesional vascularity, JZ changes, fan-shaped shadowing, and globular uterus (Table 1).

**Figure 1:**
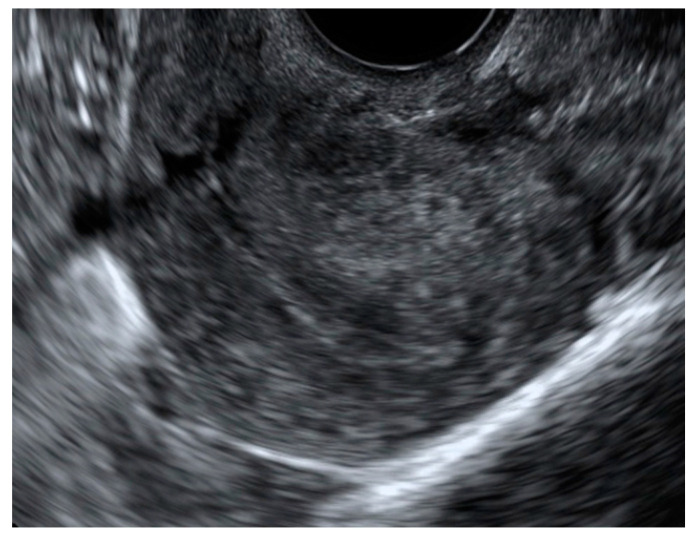
Transvaginal two-dimensional ultrasound images of a uterus affected by diffuse adenomyosis of the external myometrium and deep infiltrated endometriosis (DIE).

**Figure 2:**
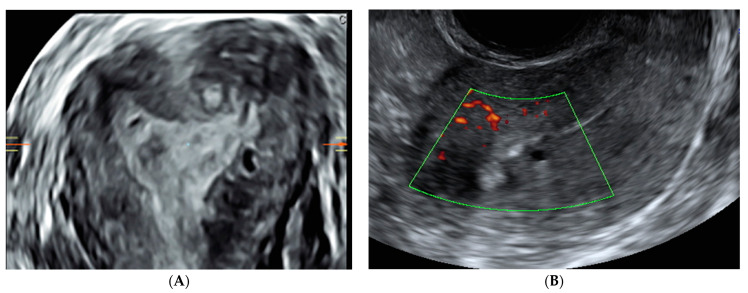
(**A**) Transvaginal three-dimensional ultrasound images of a uterus affected by subendometrial hyperechogenic lines and buds infiltrating the JZ (3D TVS). (**B**) Transvaginal two-dimensional ultrasound images of a uterus affected by focal adenomyosis of the JZ with myometrial cysts and hyperechogenic islands (2D TVS).

In addition, recently, an ultrasound sign concerning the uterus called “question sign” has been reported in the literature [27,28]. This is described when the body of the uterus is flexed backward, the fundus is bent posteriorly, and the cervix is directed frontally toward the bladder body (Figure 1). Power Doppler can be used to distinguish myometrial cysts from blood vessels and to discriminate between leiomyoma and focal adenomyosis. Uterine leiomyomas manifest circular flow along their capsules, while adenomyosis and localized adenomyomas are characterized by diffuse vessels within the lesions. Although the JZ can be visualized in a 2D image, the acquisition of a 3D volume allows for a more complete evaluation of the sagittal, transverse, and coronal planes, as shown in a standardized multiplanar view (Figure 2) [24,29,30].

Currently, detailed morphologic assessment of the JZ includes the following classifications:

- Irregular JZ with a poorly distinguishable endometrial–myometrial border;
- Disrupted JZ, possibly caused by focal or diffuse infiltration of the JZ by endometrial tissue. Uterine contractions may also give rise to apparent irregularities of the JZ or affect wall thickness;
- Alterations of the JZ due to subendometrial hyperechogenic lines and buds, ultrasound signs of focal adenomyosis infiltrating the JZ [26].

Adenomyosis should be described according to anterior, posterior, left lateral, right lateral, fundus, or cervical localization. Adenomyosis may be present in one or more locations within the uterine wall or may involve the entire myometrium. Most often, it presents as a disseminated lesion throughout the myometrium (diffuse adenomyosis) rather than being limited to localized (focal) lesions. Focal adenomyosis describes adenomyosis present in only one area of the myometrium; specifically, in focal adenomyosis, more than 25% of the lesions are surrounded by a healthy myometrium [31]. Adenomyoma describes a focal lesion with compensatory hypertrophy of the surrounding myometrium and, in rare cases, may present as a large cyst (adenomyotic cyst or cystic adenomyoma).

**Figure 3:**
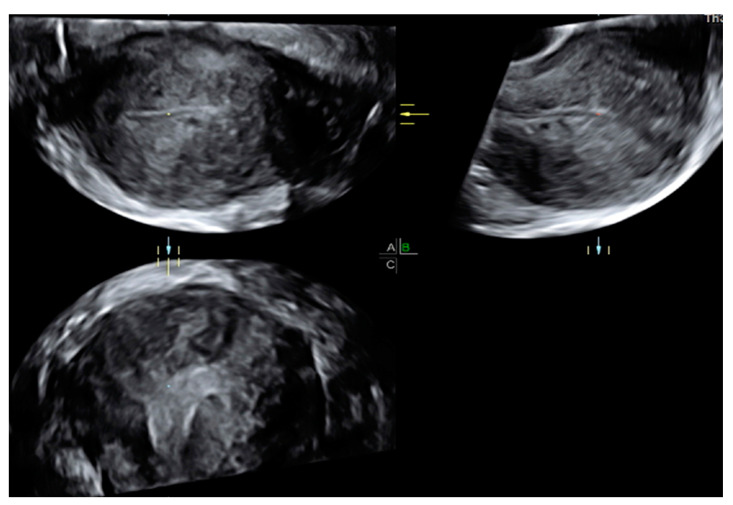
Transvaginal two-dimensional and three-dimensional ultrasound images of a uterus affected by “mixed” focal and diffuse adenomyosis of the JZ and outer myometrium.

In addition, focal and diffuse adenomyosis may coexist in the same patient in cases of “mixed adenomyosis” (Figure 3).

The depth of myometrial infiltration also varies, from cases limited to the innermost myometrium to those involving the entire myometrial thickness [20]. Adenomyosis may involve one or more uterine layers. Differentiation into two inner layers, namely the junctional zone and the external myometrium, seems practical, considering that these two layers are also embryologically different (archometrium and neometrium, respectively) [32]. The severity of adenomyosis can be classified according to MUSA criteria [26] in terms of the percentage of myometrium affected (mild, <25%; moderate, 25–50%; severe, >50%) [32].

Some studies have analyzed the association between ultrasound diagnosis of adenomyosis and endometriosis [32,33]. Certainly, the two conditions share a pathogenesis and symptoms such as dysmenorrhea, heavy menstrual bleeding, infertility, dyspareunia, and chronic pelvic pain. It has been reported that pelvic endometriosis, especially in advanced stages, is also strongly associated with adenomyosis [29,34,35].

Lazzeri et al. confirmed the strong association between adenomyosis diagnosed by TVS and deep endometriosis diagnosed and treated surgically. In this study, the incidence of adenomyosis in patients with deep infiltrating endometriosis was 48.7%. It was shown that the ultrasound “question sign” of the uterus was strongly correlated with posterior deep infiltrating endometriosis [27,28]. It seems that this type of adenomyosis, mainly of the external myometrium, is caused by the external invasion of deep endometriosis; therefore, it often appears as separate from the JZ, while adenomyosis involving the JZ originates from the endometrium. These features support the pathogenetic theories underlying adenomyosis [25,36]. Adenomyosis is a heterogeneous condition, and advancements in imaging allow for the description of the following characteristics of the disease that could have an important clinical impact in terms of symptoms and pregnancy outcomes: ultrasonographic features, localization, type, myometrial infiltration, and grade (Table 1).

### 2.2. Diagnosis by MRI

Several studies have compared the accuracy of TVS and MRI regarding the diagnosis of adenomyosis [17,18,20,22]. Three recent meta-analyses and systematic reviews showed similar sensitivity for TVS (72–79%) and MRI (77–78%) [21,22,23]. However, MRI shows higher specificity (88–93%) than TVS (78–81%). Overall, these results show that both TVS and MRI are highly accurate. The choice between TVS and MRI depends on several factors, including the availability of imaging technology, operator experience, patient-related contraindications, and the preference of the referring physician. TVS is widely available and well tolerated by most patients. Therefore, ultimately, the choice of imaging modality is left to the context of individual patients and healthcare provider preferences and defined by resource availability [37]. Recently, the group of Bazot et al. suggested a new classification of adenomyosis diagnosed by MRI according to the extension of the disease in the internal myometrium (focal: superficial and diffuse based on JZ hypertrophy; external myometrium: posterior or anterior based on localization; adenomyoma: submucosal, intramural, or subserosal according to localization) [38].

Regarding diagnosis by MRI, Kobayashi’s classification is also of meritorious importance, based on the following five radiologic signs: the area affected by the disease (inner and/or outer myometrium), the pattern (diffuse or focal), the volume (<1/3, <2/3, or >2/3 of the uterine wall), the localization (anterior, posterior, right or left lateral, or fundic), and the possible presence of concomitant pathology (endometriosis, myomas, others) [39].

Methods of 2D and 3D transvaginal ultrasound have now achieved a high level of accuracy, and many authors have reported considerable agreement between ultrasound diagnosis of adenomyosis and MRI. TVS can assess the type and severity of adenomyosis and may be useful in selecting and evaluating the effectiveness of medical and surgical management, as well as the possible relationship between adenomyosis and infertility.

## 3. Therapy for Adenomyosis

### 3.1. Medical Therapies for Adenomyosis

For patients with adenomyosis experiencing dysmenorrhea, non-steroidal anti-inflammatory drugs (NSAIDs) have proven effective in managing pain by reducing prostaglandin production [40]. NSAIDs and other pain relievers remain the primary treatment for women with adenomyosis who wish to conceive. Initially designed for long-term contraception, the levonorgestrel-releasing intrauterine system (LNG-IUS) has also been used to manage dysmenorrhea and menorrhagia [41] LNG-IUS is highly effective in reducing uterine volume, pain, and abnormal uterine bleeding (AUB), making it the first-line medical treatment for adenomyosis. [42] For patients with a lower burden of adenomyosis, LNG-IUS significantly improves health-related quality of life (QoL), particularly in managing symptoms such as dysmenorrhea and AUB, and improves hemoglobin levels [43].

Although data on the efficacy of estrogen–progestin contraceptives specifically for adenomyosis treatment are limited, these contraceptives are effective as primary treatments for heavy menstrual bleeding and dysmenorrhea [44]. A randomized clinical trial (RCT) with 57 patients diagnosed with adenomyosis showed that a six-month treatment with either combined oral contraceptive pill (COC)or LNG-IUS reduced pain and bleeding, with larger reductions observed in the LNG-IUS group [45]. COCs work by inhibiting follicle-stimulating and luteinizing hormones, thereby suppressing follicular growth and endometrial proliferation, which relieves AUB, dysmenorrhea, and chronic pelvic pain. However, the impact on adenomyotic lesions and uterine volume reduction is unclear.

Dienogest, a selective synthetic oral progestin, has effectively improved dysmenorrhea [46]. In a pilot study conducted by Kazuaki et al., patients with adenomyosis were administered oral dienogest, resulting in significant decreases in the visual analog scale (VAS) scores for dysmenorrhea, chronic pain, and dyspareunia but no relevant effects on uterus size or adenomyotic lesions [47]. A retrospective cohort study found that long-term use of dienogest for more than two years may lead to a significant decrease in uterine size, indicating its potential as a tolerable long-term treatment option for adenomyosis [47]. A recent RCT confirmed that dienogest significantly decreases patient pain scores, improves QoL, and is well-tolerated as a long-term treatment option [48].

Gonadotropin-releasing hormone (GnRH) analogs (both agonists and antagonists) are pivotal in managing adenomyosis due to their ability to downregulate gonadotropin release, leading to reduced estrogen levels and subsequent shrinkage of the uterus, alleviating associated pain [49]. In a systematic review of RCTs by Brown et al., GnRH agonist therapy was found to be superior to no treatment and placebo [50]. GnRH agonists such as leuprolide acetate have shown promise in inducing the regression of adenomyotic lesions and alleviating chronic pelvic pain [51]. Notably, Mansouri et al. reported the regression of adenomyotic lesions and resolution of chronic pelvic pain with leuprolide acetate treatment [52]. Despite the positive effects on adenomyosis, treatment with GnRH agonists of patients affected by adenomyosis undergoing in vitro fertilization (IVF) treatments seems to have no positive effects [53].

Conversely, GnRH antagonists (e.g., linzagolix and elagolix), represent an alternative treatment option by immediately antagonizing GnRH receptors in the pituitary gland, inhibiting gonadotropin secretion without the initial flare-up effect seen with agonists [54,55]. This mechanism reduces uterine size and symptoms, with potential advantages over agonists in maintaining adequate estradiol levels to prevent bone demineralization and estrogen deprivation symptoms.

Aromatase inhibitors are crucial in managing adenomyosis by inhibiting estrogen production, disrupting the hormonal milieu that promotes disease progression. Aromatase P450, an enzyme crucial for estrogen synthesis, is targeted by these inhibitors to mitigate hormonal environments conducive to adenomyosis progression [56]. In an RCT, both aromatase inhibitors (letrozole) and GnRH agonists (goserelin) effectively reduced both uterine and adenomyoma volumes [57]. Combining aromatase inhibitors with GnRH analogs demonstrated substantial uterine volume reduction, highlighting their potential synergistic benefits [58]. Women with severe adenomyosis would benefit from letrozole or a combination of GnRHs plus letrozole before receipt of treatment with assisted reproductive technology [53].

Selective progesterone receptor modulators (SPRMs) are synthetic steroids derived from norethindrone and designed to interact selectively with progesterone receptors, either activating or repressing gene transcription [59]. SPRMs such as ulipristal acetate (UPA) and mifepristone have been shown to effectively reduce uterine fibroid size, halt endometrial bleeding, and suppress luteinizing hormone peaks while maintaining normal follicle-stimulating hormone levels. In an RCT, UPA was administered to 30 women diagnosed with adenomyosis, resulting in significant decreases in pain symptoms and pictorial blood loss assessment scores [60].

Mifepristone offers affordability and a low-risk profile, making it advantageous for long-term medical therapy in adenomyosis [61]. Wang et al. reported an increase in caspase-3 expression in mifepristone-treated women, suggesting its potential to induce apoptosis in both eutopic and ectopic endometrial cells [62].

### 3.2. Surgical Conservative Therapy for Adenomyosis

Uterine artery embolization (UAE) is an angiographic procedure that utilizes embolic agents injected into the uterine arteries to induce ischemic necrosis in adenomyotic lesions. This technique targets the hypervascularity associated with adenomyosis causing hypoxia, ischemia, and tissue necrosis with minimal impact on surrounding tissues [63]. Kim et al. reported substantial improvements in dysmenorrhea (95.2%) and menorrhagia (95%), with 72.1% of patients showing a mean uterine volume reduction of 32.5% [64]. A meta-analysis involving 1049 patients across 30 studies reported significant symptom improvement in 83.1% of patients treated with UAE [65]. UAE is recommended for the management of symptomatic adenomyosis in patients who have completed childbearing and wish to preserve their uterus, but it is not advised for those desiring future pregnancies due to potential for adverse outcomes.

Radiofrequency ablation (RFA) is an emerging uterine-preserving treatment for adenomyosis, particularly focal adenomyosis. The procedure involves inserting electrodes into the target lesion under ultrasound guidance (laparoscopically or transcervically) and generating heat to induce thermal fixation and coagulative necrosis [66]. A systematic review reported a 94.7% rate of symptom relief with RFA and significant reductions in AUB and dysmenorrhea pain scores [67]. Additional studies have shown promising results for RFA in reducing adenomyosis volume and symptom severity [68].

High-intensity focused ultrasound (HIFU) is a non-surgical treatment option that uses ultrasound waves to thermally ablate adenomyotic lesions, causing coagulative necrosis and cell death. HIFU has been reported to result in a significant decrease in dysmenorrhea scores and the volumes of adenomyotic lesions (both focal and diffuse) [69]. It also significantly improves menorrhagia and offers sustained symptom relief. A meta-analysis revealed significant reductions in uterine volume and dysmenorrhea and an improvement in QoL post treatment. HIFU has shown superior efficacy in treating adenomyotic tissue located on the anterior uterine wall compared to the posterior wall because limited penetration of ultrasonic waves can result in suboptimal ablation for deeply situated adenomyosis [70].

Regarding fertility, the effect of HIFU is not well established. Pre- and post-treatment anti-Mullerian hormone levels showed no significant difference, suggesting that HIFU does not affect ovarian function [71]. Compared to laparoscopic excision, HIFU showed significantly higher pregnancy and natural conception rates, with a comparable effect in terms of pain and menorrhagia reduction.

A systematic review by Chen et al. assessed 557 patients seeking to conceive post HIFU treatment, reporting a pooled pregnancy rate of 53.4% and a live birth rate of 35.2% [72]. However, the significant heterogeneity among the included studies suggests the need for a cautious interpretation of these findings and highlights the necessity of further research.

Uterus-sparing surgical approaches have been developed for the treatment of adenomyosis by removing diseased tissue, reducing uterine size and alleviating clinical symptoms [73]. These methods are considered especially for patients desiring future childbearing or for whom first-line therapies have been ineffective or are contraindicated. Conservative surgical techniques have been related to marked improvements in menorrhagia and dysmenorrhea in several cases post endometrial resection, myometrial reduction, and myometrial excision [74]. A systematic review by Grimbizis et al. confirmed those results, reporting an additional increase in pregnancy rates post complete excision [75]. Recurrence risk varies depending on the radicality of the ablation, with low risk for complete excision (9%) and higher risks for partial (19%) and non-excisional techniques (32.5%) [76].

Preoperative MRI is essential to evaluate the location and extent of adenomyosis, determine the feasibility of surgery, and aid in surgical planning. Both laparoscopic and laparotomy techniques can be utilized. Conservative resection carries a higher risk of uterine rupture and abnormal placentation in subsequent pregnancies than after myomectomy or cesarean birth [77]. In 2011, Osada developed a laparoscopically assisted laparotomic technique based on extensive removal of adenomyotic tissue using preventive hemostatic methods, followed by the reconstruction of the uterine wall with a triple-flap approach [78]. Among various excisional techniques for diffuse adenomyomectomy, the Osada procedure is reported to result in the best obstetric outcomes. A meta-analysis of 12 studies found that among 364 patients who attempted to conceive, 35% were successful, with 18% miscarrying, 7% delivering preterm, and 74% having full-term births [79], with a uterine rupture rate of 0.8%.

Hysteroscopy is a minimally invasive option for adenomyotic focal lesions close to the endometrium. In an office setting, it is possible to enucleate superficial focal adenomyomas or evacuate cystic hemorrhagic lesions less than 1.5 cm in diameter using mechanical instruments or bipolar electrodes [80]. This treatment is feasible only when the lesions are recognizable by hysteroscopy, as they bulge into the endometrial cavity [81]. The traditional technique used for the enucleation of submucosal myomas with an intramural component is often adopted, although it requires careful exploration due to the lack of a distinct cleavage plane for healthy myometrial tissue identification [21].

For deeper cystic lesions localized in the intramural portion, the Spirotome can be a useful tool. Under ultrasound guidance, this device creates a channel and provides hysteroscopic access to the cystic structure, allowing for treatment by resection or bipolar coagulation.

Resectoscopic treatment is indicated for superficial adenomyotic nodules larger than 1.5 cm and diffuse superficial adenomyosis.

Furthermore, adenomyosis could change the hormonal status and cause inflammation, leading to altered endometrial receptivity and infertility. The use of hysteroscopy for examination of the uterine cavity remains a topic of debate in the IVF literature. Hysteroscopy may offer the advantage of direct visualization of the uterine cavity while providing the option to collect histological biopsy samples under visual control. Although diagnostic hysteroscopy cannot establish a definitive diagnosis of adenomyosis, the following pathological conditions of probable inflammatory status are associated with adenomyosis: an irregular endometrium with tiny openings seen on the endometrial surface; pronounced hypervascularization; an endometrial “strawberry” pattern; the appearance of fibrous, cystic intrauterine lesions (following 3–5 episodes of intramyometrial hemorrhage); and hemorrhagic cystic lesions assuming a dark blue or chocolate brown appearance [81].

## 4. Adenomyosis and Reproductive Outcomes

The effect of adenomyosis on reproductive outcomes remains a hot topic in the literature. three independent meta-analyses indicated lower pregnancy rates and higher miscarriage rates in IVF patients with adenomyosis [15]. Unfortunately, several of the studies included in the meta-analyses were biased to the diagnosis of adenomyosis not in line with the MUSA criteria [31] and the high heterogeneity of the included populations. Specifically, the potentially harmful effects of adenomyosis in cycles with autologous oocytes without preimplantation genetic tests remain unusual. A transcriptomic study demonstrated altered endometrial receptivity in women with adenomyosis relative to infertile women without adenomyosis, probably related to local inflammation. Transcriptomic analysis demonstrated a different expression of the genes involved in the mechanisms of implantation. However, adjustments in progesterone timing were not found to increase the success rate in women with adenomyosis [82].

Recently, a prospective cohort study explored the reproductive outcomes of 99 adenomyosis patients compared to 549 non-adenomyosis patients undergoing PGT-A and subsequent freeze/thaw embryo transfer (HRT-FET). In this study, no difference in live births was seen between the adenomyosis and non-adenomyosis groups [83]. However, most patients fulfilled only one MUSA criterion, whereas 17 patients had two features, and this group experienced a slight but not significant decrease in Live Birth Rate (LBR). Thus, women fulfilling one MUSA criterion only may present a less severe phenotype of adenomyosis compared to women with more features. In addition, the study did not report information about the luteal support.

Many studies have attempted to find a correlation between adenomyosis and reproductive outcomes, with contradictory results.

A recent study demonstrated that adenomyosis is associated with RPL. Specific signs of adenomyosis, such as focal adenomyosis of the JZ, seem to be important in evaluating the risk of repeat pregnancy failure (PRL) compared with patients without PRL, but no differences were found in the severity of adenomyosis between the two groups of women [84]. Another study observed a higher percentage of infertility and miscarriage in focal adenomyosis of both the outer myometrium and the JZ [85]. A recent study conducted in women with adenomyosis and donor oocytes demonstrated that although adenomyosis did not significantly reduce the odds of implantation, the direct signs of adenomyosis in the JZ and disease severity are significant risk factors for miscarriage in these patients [14]. This study highlights the importance of thorough ultrasound examination and detailed adenomyosis classification in the assessment and management of patients with infertility. Such examinations can prevent the worsening of these conditions by facilitating early diagnosis of severe dysmenorrhea in women by an expert sonographer in the field [86,87].

Another prospective cohort study of oocyte recipients suggested that adenomyosis does not significantly hamper implantation; however, the presence of adenomyosis increased the early miscarriage rate, resulting in an LBR of 36.8% in the adenomyosis group of patients (n = 114) compared to 43.9% in the non-adenomyosis group (n = 114) [14]. Although the difference was 7% in favor of non-adenomyosis, the difference did not reach statistical significance with the sample size. JZ involvement and adenomyosis severity appear to be important risk factors for miscarriage. The study demonstrated the importance of accurate ultrasound examination and classification of adenomyosis for effective counseling of previous IVF patients. In support of these findings, a recent retrospective HRT-FET cohort study including a total of 3503 first blastocyst transfer patients, of whom 140 were diagnosed with adenomyosis, showed that adenomyosis significantly decreased clinical pregnancy rates (aOR 0.62, 95% CI: 0.39–0.98, *p* = 0.040) and live birth rates (aOR 0.46, 95% CI: 0.27–0.75, *p* = 0.003) and significantly increased miscarriage rates (aOR 2.13, 95% CI: 0.98–4.37, *p* = 0.045) [88]. Women with adenomyosis have an increased risk of miscarriage, even using euploid embryos.

Among women with adenomyosis, the freeze-all strategy represents the primary choice. From a physiological point of view, the concept of a freeze-all strategy makes sense, as ovarian stimulation in IVF patients results in multi-follicular development, leading to high circulating and local estradiol levels, which, per se, create progesterone resistance, in line with the hypothesis that hyperestrogenism has a detrimental effect on adenomyosis [56].

GnRH analogs have an antiproliferative and noninflammatory effect on the myometrium and induce a local hypoestrogenic effect through the central downregulation and suppression of gonadotropin secretion. Treatment with GnRH agonist is considered useful before frozen embryo transfer. However, the number of administrations necessary for embryo transfer remains unclear. In a large retrospective cohort study, the live birth rate increased after pretreatment with an ultra-long GnRH agonist compared with a long GnRH agonist [89]. In a similar retrospective cohort study, an ultra-long GnRH agonist protocol was also associated with an increased live birth rate and clinical pregnancy rate with fresh transfer relative to a long agonist protocol [90]. The impact of GnRH agonist pretreatment on the live birth rate and clinical pregnancy rate may be different for fresh and frozen cycles [91]. Treatment with low-dose letrozole is an alternative to GnRH agonist treatment, with comparable effects in terms of improving the symptoms and sonographic features of adenomyosis in women awaiting IVF [92]. Women with severe adenomyosis and without suppression of the serum estradiol after 1–5 months of downregulation with GnRHs were treated with a combination of GnRH downregulation for 2 months and 2.5 mg of letrozole twice per day for 21 days before stimulation with hormone replacement therapy [53].

The aromatase inhibitors mainly exert local effects, blocking excess estradiol production in the endometrium. Adenomyosis is a hormonally active disease that can be treated by controlling estrogen production, thus, progesterone receptor affinity [92]. Despite the positive effects on adenomyosis, GnRH agonist treatment in patients affected by adenomyosis undergoing in vitro fertilization (IVF) treatments seems to have no positive effects [15]. It would also be interesting to evaluate the effect of GnRH antagonists (e.g., linzagolix and elagolix) without the initial flare-up effect seen with agonists and their impact on reproductive outcomes [54,55].

## 5. Conclusions and Future Directions

Considering the included publications, with subsequent evidence of improvement in our knowledge on the pathogenesis of adenomyosis, we can consider a link between adenomyosis and infertility/subfertility. The biological basis for a suspected negative impact of adenomyosis on fertility includes altered endometrial receptivity and dysregulation of local hormonal metabolism, leading to a hyperestrogenic milieu.

An important revolution in this topic would be an accurate diagnosis of the disease. Recent advancements in 23 and 3D TVS imaging have led to early diagnosis with high sensitivity and specificity compared to MRI and/or histologic findings, ranging from 7 to 88% and 67 to 93%, respectively [17,18,19,20,21,22,23].

In conclusion, the effect of adenomyosis is still debated in the literature. Three independent meta-analyses indicated a reduced pregnancy rate and increased miscarriage rates in IVF patients with adenomyosis [93]. On the other hand, no difference in live births was demonstrated between adenomyosis and non-adenomyosis groups [84].

Certainly, the freeze-all strategy should be considered the primary choice in patients with adenomyosis. The treatment of adenomyosis could be crucial for reproductive outcomes. GnRH analogs, GnRH antagonists, low-dose letrozole treatment, and their combination have antiproliferative effects on the myometrium and induce a local hypoestrogenic effect through the central downregulation and suppression of gonadotropin secretion. For this reason, they are considered useful treatments before frozen embryo transfer. Despite this, there is no accordance and evidence-based medicine with RCT studies to evaluate the effect of these pharmacological treatments before embryo transfer on reproductive outcomes in terms of LBR.

Most studies on this issue are characterized by substantial qualitative and quantitative heterogeneity. This makes a reliable assessment of the available evidence difficult, and caution should be exercised when attempting to derive clinical indications that may affect the reproductive outcomes of the women affected by adenomyosis.

## Figures and Tables

**Table 1:** Ultrasonographic features based on MUSA (2022) and the characteristics describing adenomyosis.

Ultrasonographic Characteristics of Adenomyosis	Ultrasound
**Ultrasonographic features of adenomyosis**	Myometrial cyst * Hyperechogenic islands * Echogenic subendometrial lines and buds * Globular uterus ** Asymmetrical myometrial thickening ** Fan-shaped shadowing ** Translesional vascularity ** Irregular JZ ** Interrupted JZ **
**Localization**	Anterior Posterior Lateral (right, left) Fundal Cervical
**Type of adenomyosis**	Diffuse Focal Adenomyoma Mix (diffuse and focal)
**Myometrial infiltration**	External myometrium Internal myometrium or junctional zone (JZ)
**Grade**	Mild (<25% of myometrium) Moderate (25–50% of myometrium) Severe (> 50% of myometrium)

* Direct features; ** indirect features.
